# Evaluation of the invasiveness of pure ground-glass nodules based on dual-head ResNet technique

**DOI:** 10.1186/s12885-024-12823-4

**Published:** 2024-09-02

**Authors:** Dengfa Yang, Yang Yang, MinYi Zhao, Hongli Ji, Zhongfeng Niu, Bo Hong, Hengfeng Shi, Linyang He, Meihua Shao, Jian Wang

**Affiliations:** 1 Department of Radiology, Taizhou Municipal Hospital, Taizhou, 318000 China; 2 Department of Radiology, The First Affiliated Hospital of Bengbu Medical University, Bengbu, 233004 China; 3 Jianpei Technology, Hangzhou, 311202 China; 4 Department of Radiology, Sir Run Run Shaw Hospital, Zhejiang University School of Medicine, Hangzhou, 310009 China; 5 Department of Radiology, Anqing Municipal Hospital, Anqing, 246004 China; 6 Department of Radiology, Tongde Hospital of Zhejiang Province, Hangzhou, 310012 China

**Keywords:** Deep learning, Tumor invasiveness, Pure ground-glass nodule, Computed tomography

## Abstract

**Objective:**

To intelligently evaluate the invasiveness of pure ground-glass nodules with multiple classifications using deep learning.

**Methods:**

pGGNs in 1136 patients were pathologically confirmed as lung precursor lesions [atypical adenomatous hyperplasia (AAH) and adenocarcinoma in situ (AIS)], minimally invasive adenocarcinoma (MIA), or invasive adenocarcinoma (IAC). Four different models [EfficientNet-b0 2D, dual-head ResNet_3D, a 3D model combining three features (3D_3F), and a 3D model combining 19 features (3D_19F)] were constructed to evaluate the invasiveness of pGGNs using the EfficientNet and ResNet networks. The Obuchowski index was used to evaluate the differences in diagnostic efficiency among the four models.

**Results:**

The patients with pGGNs (360 men, 776 women; mean age, 54.63 ± 12.36 years) included 235 cases of AAH + AIS, 332 cases of MIA, and 569 cases of IAC. In the validation group, the areas under the curve in detecting the invasiveness of pGGNs as a three-category classification (AAH + AIS, MIA, IAC) were 0.8008, 0.8090, 0.8165, and 0.8158 for EfficientNet-b0 2D, dual-head ResNet_3D, 3D_3F, and 3D_19F, respectively, whereas the accuracies were 0.6422, 0.6158, 0.651, and 0.6364, respectively. The Obuchowski index revealed no significant differences in the diagnostic performance of the four models.

**Conclusions:**

The dual-head ResNet_3D_3F model had the highest diagnostic efficiency for evaluating the invasiveness of pGGNs in the four models.

**Supplementary Information:**

The online version contains supplementary material available at 10.1186/s12885-024-12823-4.

## Introduction

Lung cancer is the leading cause of cancer-related mortality globally, and the most common subtype is lung adenocarcinoma [1, 2]. The new classification of lung tumors issued by WHO in 2021 classified lung epithelial tumors as lung precursor lesions [atypical adenomatous hyperplasia (AAH) and adenocarcinoma in situ (AIS)], minimally invasive adenocarcinoma (MIA), or invasive adenocarcinoma (IAC) [3]. Studies have confirmed that persistent pure ground-glass nodules (pGGNs) can be pathologically diagnosed as pre-invasive carcinoma or IAC [4, 5], and 30–40% of patients with resected pGGNs are IAC [6, 7]. Therefore, the imaging features of different histopathological subtypes manifesting as pGGNs on computed tomography (CT) overlap significantly.

It is generally known that lung precursor lesions can be followed up without urgent surgical resection. In addition, MIA can be followed up without surgery or treated by partial resection via lung lobectomy. Meanwhile, IAC should be treated with an appropriately extended scope of resection or complete lobectomy if necessary, and lymph node dissection is required [8–10]. AIS and MIA have a 5-year disease-free survival rate of 100% [11, 12], whereas that for IAC ranges from 33.3 to 99.0% depending on the major histological subtype [13]. Therefore, it is extremely important to accurately distinguish lung precursor lesions, MIA, and IAC in the new classification to select the best treatment to optimize patient prognosis.

Thus far, studies on the infiltration of pGGNs based on morphological changes on CT have revealed that a larger diameter and mass, higher attenuation value, irregular shape, lobulation, spiculation, cavitation, and internal vascular changes are closely related to the degree of infiltration [5, 7, 14–19]. However, it remains difficult to clarify the invasiveness of pGGNs by CT. The possible reasons are as follows: (1) measurements of size and assessments of signs are susceptible to multiple factors (e.g., reproduction of measurement, scanning parameter, reconstruction algorithm, observer experience); (2) some signs have a low incidence in pGGNs and the ability to identify lung precursor lesions is limited; and (3) most features in pre-invasive adenocarcinoma and IAC overlap.

In recent years, increasing numbers of scholars have focused on the application of radiomics for clarifying the invasiveness of pGGNs. Fan et al. [20]. reported that radiomics had good predictive performance in differentiating IAC and non-invasive adenocarcinoma from ground-glass nodules (GGNs). In the external validation group, the area under the curve (AUC) and accuracy (ACC) were 0.936 and 0.881, respectively, suggesting that radiomics could be used as a non-invasive modality for determining follow-up and treatment strategies for lung adenocarcinoma. Other studies demonstrated the good performance of radiomics in predicting IAC with pGGNs and pGGNs invading the pleura, and the AUCs were 0.72 and 0.862, respectively, in the validation group [21, 22]. The radiomic nomogram is helpful for evaluating the invasiveness of pGGNs. It also provides valuable reference information for follow-up and elective surgical treatment. However, radiomic features are derived from manual segmentation, and 3D tumor segmentation is a complex and time-consuming process. At the same time, small blood vessels and bronchi must be avoided during the procedure, but the remaining blood vessels can affect the accuracy of some features. Therefore, both radiological and radiomic models have limitations such as subjective judgment, manual segmentation, and time and labor consumption, which affect the prediction results.

To date, some studies have revealed that artificial intelligence, including convolutional neural networks, is useful for the detection, characterization, and identification of GGNs on CT [23, 24]. Zhao et al. [25] reported that the 3D DenseSharp Network automatically predicted the invasiveness of subcentimeter GGNs (three-category classification) with an ACC of 0.641 and achieved better classification performance than radiologists. Gong et al. [26]. used the deep residual learning network to predict the invasiveness of GGNs (two-category classification: pre-invasive lesions and IAC), and the AUC was 0.92 ± 0.03, reflecting an improved prediction performance for IAC. Lv et al. [27] reported that 3D conventional networks automatically predicted the invasiveness of GGNs (two-category classification), and the AUC was 0.862, similar to that for intraoperative frozen-section analysis. At present, research has used simple 3D deep-learning technology to explore the invasiveness of GGNs.

To the best of our knowledge, no prior study applied a deep learning technique to assess the degree of infiltration of pGGNs. The deep learning network was adopted in the study, which can automatically identify, monitor, qualitative and prognostic judgment of GGNs on CT, with high repeatability, good stability and short time, and can effectively overcome the limitations of subjective judgment, artificial segmentation, time and energy consumption. Therefore, based on EfficientNet-b0 and dual-headed ResNet networks and combinations of different clinical features, four different models were constructed to evaluate the invasiveness of pGGNs and provide a basis for accurate diagnosis and treatment.

## Materials and methods

### Patient data

**Fig. 1:**
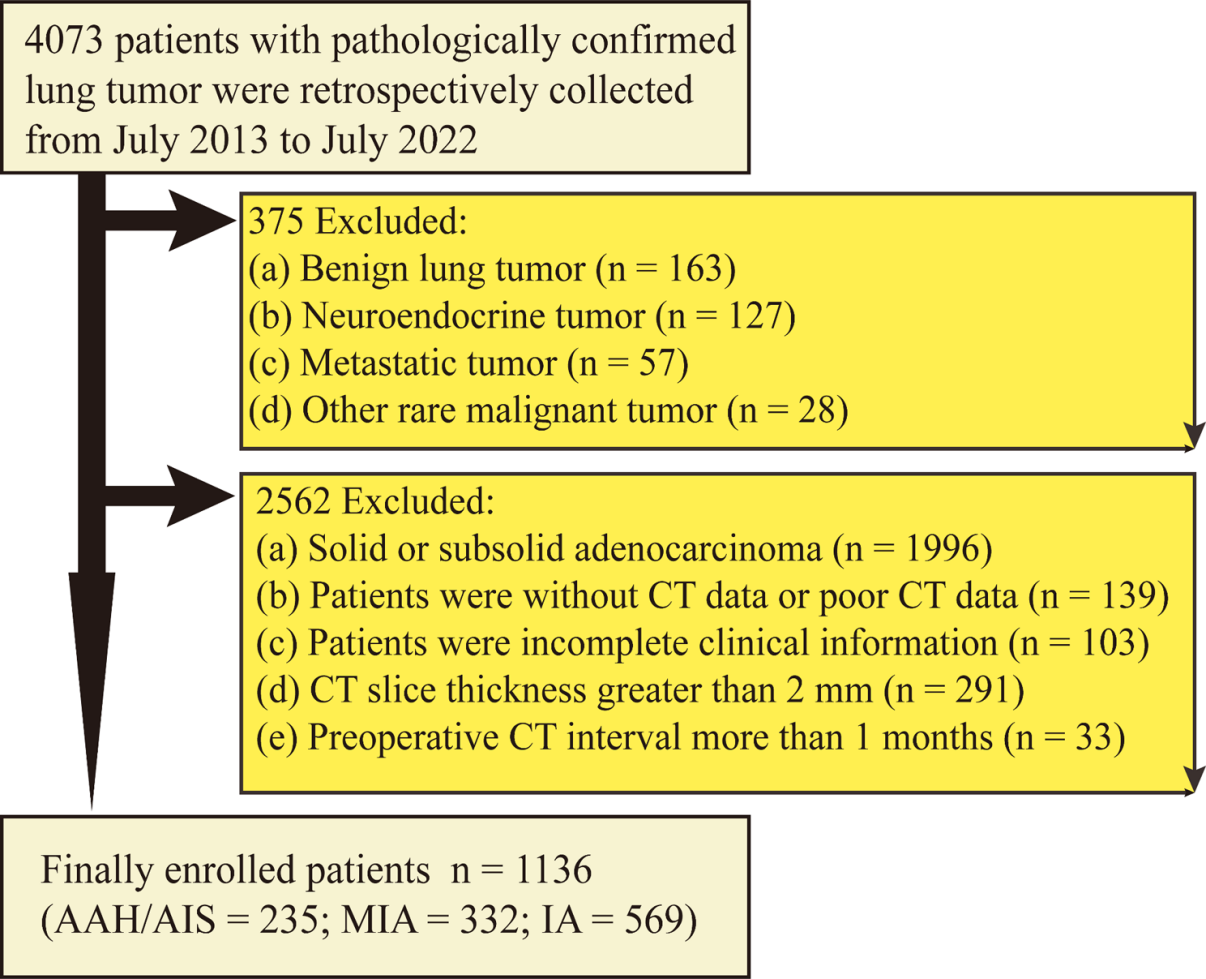
Patient flow chart

In total, 4073 patients with surgically confirmed lung tumors were identified from July 2013 to July 2022. The inclusion criteria were as follows: (1) pathological confirmation of the lesion as IAC, MIA, AIS, or AAH; (2) chest CT revealed pGGNs without solid parts; (3) use of thin-slice chest CT (thickness ≤ 1.5 mm); and (4) no treatment before surgery. The exclusion criteria included a duration of more than 1 month between CT and surgery, incomplete clinical data, and poor CT image quality. Finally, 1136 patients were included in the study. If multiple pGGNs were surgically treated in one patient, one nodule was selected as the study object with infiltration as the main factor and size as the auxiliary factor. Details of the inclusion and exclusion criteria are presented in Fig. 1.

### CT inspection methods

All patients underwent routine CT. The CT scanners were as follows: Somatom Emotion 16 (Siemens, Erlangen, Germany), Definition Flash (Siemens), Force CT (Siemens), Optima CT680 (GE Healthcare, Chicago, IL, USA), and Light Speed 64 (GE Healthcare). The CT scanning parameters were as follows: tube voltage, 100–120 kV; tube current, 200–280 mA; pitch, 0.8–1.0; collimator, 0.6–0.65; and matrix, 512 × 512. The scanning layer and reconstruction thickness were 5 and 1–2 mm, respectively, and standard reconstruction algorithms were used.

### Image processing and model construction

**Fig. 2:**
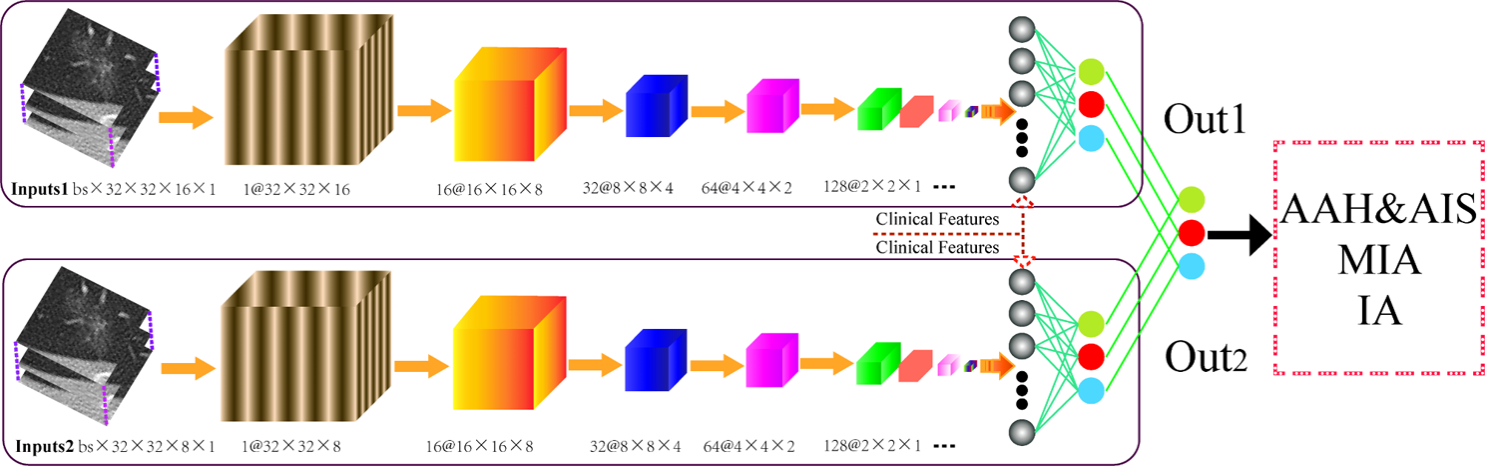
The entire structure of the 3D model dual-head ResNet network

In this study, the EfficientNet-b0 and dual-head ResNet networks were used. The entire structure of the 3D model dual-head Res2Net network is presented in Fig. 2. The specific image processing and model construction are presented in the Supplementary file 1.

### Clinical data and imaging analysis

Clinical data, including sex, age, smoking status, operation status, underlying diseases, tumor markers, and pulmonary diseases, were obtained from the patients’ medical records. CT images were evaluated by two senior physicians with experience in chest diseases (with 12 and 18 respective years of experience), who agreed on the results. CT data were analyzed for the following variables: distribution, lobulation, spiculation, cystic airspace, air bronchogram type, pleural contact type, long diameter (LD), short diameter (SD), maximum CT attenuation (CTmax), minimum CT attenuation (CTmin), mean CT attenuation (CTmean), and standard deviation of CT attenuation (CTsd). In addition, LD and SD were measured for each lesion on the axial image. The CT values were the mean values of multiple measurements taken by two physicians. Pleural contact was divided into three types: direct contact (Type I), pleural tag (Type II), and Type I + Type II (Type III). Four models including the 2D model, 3D model, 3D model combining three clinical features (sex, age, smoking status; 3D_3F model), and 3D model combining 19 clinical features (sex, age, smoking status, operation status, underlying diseases, tumor markers, pulmonary diseases, distribution, lobulation, spiculation, cystic airspace, air bronchogram type, pleural contact type, LD, SD, CTmax, CTmin, CTmean and CTsd).

### Statistical analysis

SPSS 25.0 software was used for statistical analysis. Qualitative data were presented as the frequency (component ratio), and the χ^2^ test was used for intergroup comparisons. Pearson’s correlation analysis was selected when the minimum expected value was greater than 5, and Spearman’s correlation analysis was selected when the minimum expected value was less than or equal to 5 and greater than 1. The Shapiro–Wilk test was used to determine whether the quantitative data were normally distributed. Binary quantitative data with a normal distribution were presented as the mean ± standard deviation, and comparisons between the groups were performed using an independent-samples *t*-test. Tripartite quantitative data with a normal distribution were presented as the mean median (interquartile range), and comparisons between the groups was performed by ANOVA. The quantitative data of two categories that did not conform to a normal distribution were presented as the median (interquartile range), and the Mann–Whitney U test was used for intergroup comparisons. The quantitative data of three categories that did not conform to a normal distribution were presented as the median (interquartile range), and intergroup comparisons were performed using the Kruskal–Wallis H test (Welch test). The diagnostic performance of artificial intelligence models was evaluatedby the area under the receiver operating characteristic (ROC) curve (AUC), Accuracy(ACC) and other evaluation metrics, such as Recall, Precision, F1-Score. Python software was used to construct the 2D and 3D models and evaluate model efficiency.

## Results

### General clinical data of the included patients

The clinical data of the 1136 patients are presented in Table 1. The numbers of patients in the training set with AAH + AIS, MIA, and IA were 155, 248, and 392, respectively, whereas the numbers in the validation set were 80, 84, and 177, respectively. Concerning the general clinical data, the mean patient age significantly differed among the groups in both the training and validation sets (both *P* < 0.05). The incidence of IAC increased with increasing age. In addition, the sex ratio, prevalence of underlying diseases, and tumor marker levels significantly differed among the groups in the training set (all *P* < 0.05). The distribution of each index was balanced in the training and validation sets.

**Table 1:** The clinical characteristics of 1136 patients in both training and validation sets

Characteristic	Training set			*P*_t_	Validation set			*P*_v_	*P*_t&v_
	AAH/AIS (*n* = 155)	MIA (*n* = 248)	IA (*n* = 392)		AAH/AIS (*n* = 80)	MIA (*n* = 84)	IA (*n* = 177)		
Sex (Male)^†^	41(26.5)	69(27.8)	145(37.0)	0.013	22(27.5)	22(26.2)	63(35.6)	0.216	0.817
Mean age (y)*	53(46,61)	53(44,61)	58(48,66)	<0.001	54(47,62)	53(48,62)	59(50,67)	0.005	0.129
Smoking status				0.111^‡^				0.253^‡^	0.270^†^
Never smoker	140(90.3)	235(94.8)	354(90.3)		71(88.8)	79(94.0)	159(89.8)		
Current smoker	13(8.4)	8(3.2)	26(6.6)		7(8.8)	1(1.2)	10(5.6)		
Former smoker	2(1.3)	5(2.0)	12(3.1)		2(2.5)	4(4.8)	8(4.5)		
Operation status^†^				0.833				0.306	0.788
No	117(75.5)	190(76.6)	305(77.8)		58(72.5)	61(72.6)	141(79.7)		
Yes	38(24.5)	58(23.4)	87(22.2)		22(27.5)	23(27.4)	36(20.3)		
Underlying diseases^†^				0.001				0.614	0.407
No	120(77.4)	197(79.4)	267(68.1)		56(70.0)	64(76.2)	129(72.9)		
HE/HL/HC	24(15.5)	38(15.3)	63(16.1)		13(16.3)	11(13.1)	22(12.4)		
Other	7(4.5)	4(1.6)	26(6.6)		5(6.3)	7(8.3)	11(6.2)		
Mixed	4(2.6)	9(3.6)	36(9.2)		6(7.5)	2(2.4)	15(8.5)		
Tumor marker^†^				0.037				0.654	0.040
Normal	140(90.3)	221(89.1)	327(83.4)		67(83.8)	66(78.6)	149(82.5)		
Abnormal	15(9.7)	27(10.9)	65(16.6)		13(16.3)	18(21.4)	31(17.5)		
Pulmonary disease				0.090^♀^				0.150^♀^	0.556^‡^
No	137(88.4)	222(89.5)	323(82.4)		70(87.4)	79(94)	146(82.5)		
Emphysema/Bullae	7(4.5)	13(5.2)	31(7.9)		4(5.0)	2(2.4)	11(6.2)		
VPI	9(5.8)	8(3.2)	30(7.7)		5(6.3)	3(3.6)	17(9.6)		
Other	1(0.6)	4(1.6)	2(0.5)		0	0	1(0.6)		
Mixed	1(0.6)	1(0.4)	6(1.5)		1(1.3)	0	2(1.1)		

*Note*—Unless otherwise indicated, the data are qualitative variables, the number of patients are outside the brackets, the percentages are inside the brackets, and the statistical values are Pearson’s χ^2^ test (†), Spearman’s χ^2^ test (^‡^) and Fisher’s exact test (^♀^);AAH = atypical adenomatous hyperplasia, AIS = adenocarcinoma in situ, MIA = minimally invasive adenocarcinoma, IA = invasive adenocarcinoma, HE = hypertension, HL = hyperlipidemia, HC = hyperglycemia, VPI = Ventilation perfusion imbalance

^†^Pearson was used for chi-square test results with minimum expected value greater than 5

^‡^Spearman was selected for the minimum expected value greater than 1 and less than 5 in chi-square test results

^♀^Fisher’s was used for chi-square test results with minimum expected value greater than 1

*Data do not conform to normal distribution, the median are outside parentheses, while the lower quartile and the upper quartile are in parentheses, and the statistical values of two or three group are obtained from Mann-Whitney U test or Kruskal-wallis H test, respectively

### CT findings of the study cohort

**Fig. 3:**
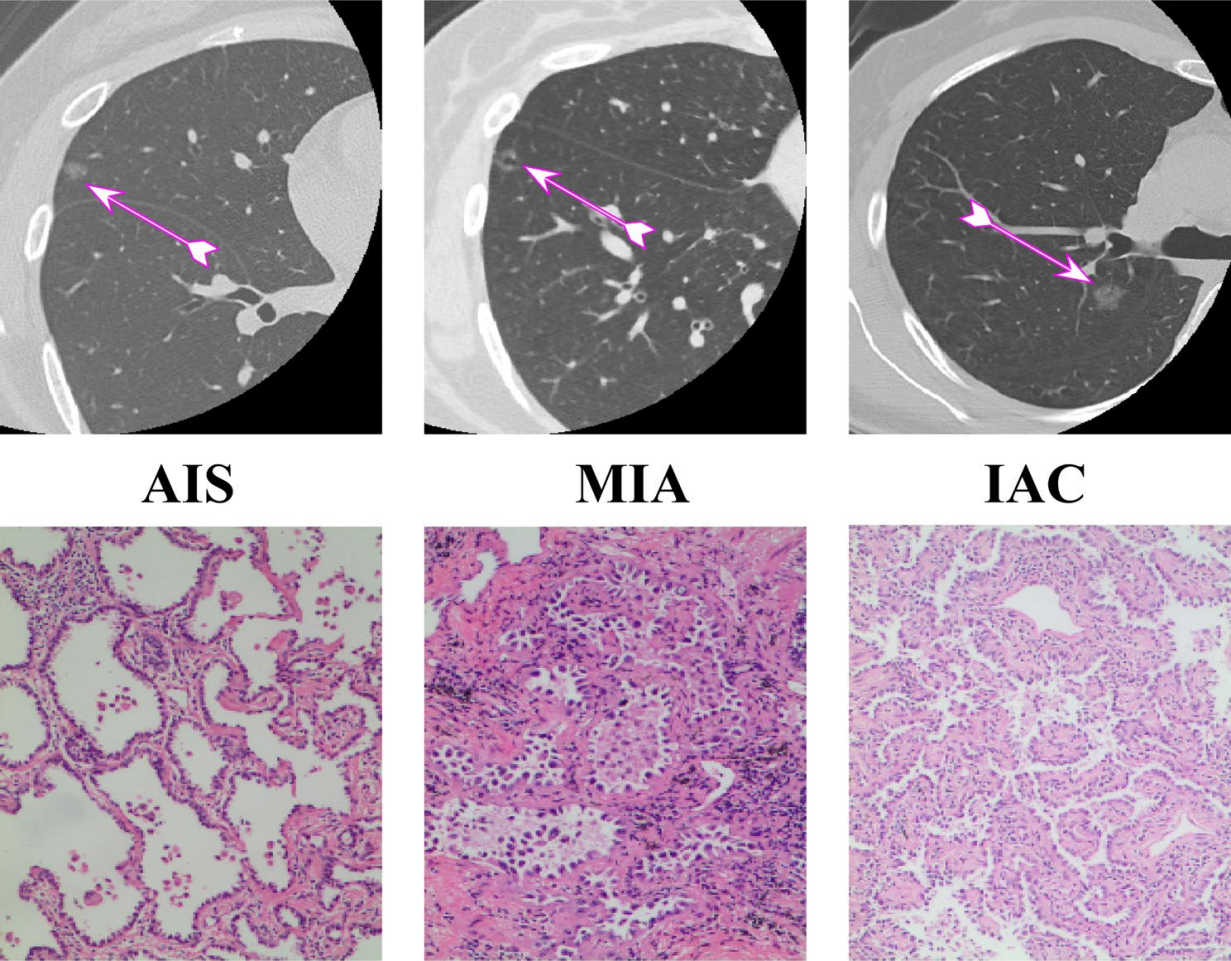
CT images showed three pure ground glass nodules (arrows), pathologically representing AIS, MIA and IAC, respectively. Among them, cystic airspace was seen on the MIA nodule, and lobulation on the IAC nodule

The CT findings of the 1136 patients are presented in Table 2. Lobulation, spiculation, air bronchogram type, LD, SD, CTmax, CTmin, CTmean, and CTsd significantly differed among the groups in the training and validation sets (all *P* < 0.05) (Fig. 3). The lesion distribution, cystic airspace, and pleural contact type significantly differed among the groups in the training set (all *P* < 0.05). The distribution of each index was balanced in the training and validation sets.

**Table 2:** Th**e** CT features of 1136 patients in both training and validation sets

Feature	Training set			*P*_t_	Validation set			*P*_v_	*P*_t&v_
	AAH/AIS (*n* = 155)	MIA (*n* = 248)	IA (*n* = 392)		AAH/AIS (*n* = 80)	MIA (*n* = 84)	IA (*n* = 177)		
Location^†^				0.011				0.301	0.438
Right upper lobe	56(36.1)	85(34.3)	149(38.0)		31(38.8)	22(26.2)	57(32.2)		
Right middle lobe	15(9.7)	28(11.3)	15(3.8)		11(13.8)	10(11.9)	13(7.3)		
Right lower lobe	26(16.8)	43(17.3)	59(15.1)		9(11.3)	18(21.4)	31(17.5)		
Left upper lobe	47(30.3)	61(24.6)	116(29.6)		22(27.5)	24(28.6)	48(27.1)		
Left lower lobe	11(7.1)	31(12.5)	53(13.5)		7(8.8)	10(11.9)	28(15.8)		
Lobulation^†^	25(16.1)	62(25.0)	185(47.2)	<0.001	10(12.5)	20(23.8)	95(53.7)	<0.001	0.429
Spiculation^†^	4(2.6)	19(7.7)	58(14.8)	<0.001	5(6.3)	9(10.7)	35(19.8)	0.009	0.040
Cystic airspace^†^	8(5.2)	14(5.6)	53(13.5)	<0.001	9(11.3)	9(10.7)	30(16.9)	0.283	0.021
Air bronchogram type				<0.001^‡^				<0.001^♀^	0.053^‡^
No	155(100)	239(96.4)	364(92.9)		80(100)	84(100)	158(89.3)		
Without BD	0	2(0.8)	5(1.3)		0	0	2(1.1)		
With BD	0	7(2.8)	23(5.9)		0	0	17(9.6)		
Pleural contact type*				<0.001^♀^				0.077^♀^	0.297^†^
No	117(75.5)	156(62.9)	247(63.0)		58(72.5)	57(67.9)	108(61.0)		
Type I	38(24.5)	89(35.9)	122(31.1)		20(25.0)	27(32.1)	66(37.3)		
Type II	0	3(1.2)	20(5.1)		2(2.5)	0	2(1.1)		
Type III	0	0	3(0.8)		0	0	1(0.6)		
LD (mm)*	8(7,10)	8(7,11)	12(9,16)	<0.001	8(7,10)	8(7,10)	13(10,17)	<0.001	0.213
SD (mm)*	7(5,8)	7(5,9)	9(7,12)	<0.001	6(5,8)	7(5,8)	10(7,13)	<0.001	0.395
CT_max_ value (HU)*	-428(-518,-268)	-283(-402,-119)	-152(-294,-23)	<0.001	-433(-542,-285)	-257(-355,-176)	-147(-272,-25)	<0.001	0.900
CT_min_ value (HU)*	-641(-739,-551)	-536(-631,-414)	-484(-611,-340,)	<0.001	-661(-732,-550)	-520(-658,-392)	-505(-616,-369,)	<0.001	0.257
CT_mean_ value (HU)*	-527(-605,-407)	-409(-501,-280)	-317(-447,-194)	<0.001	-521(-616,-436)	-387(-462,-297)	-329(-433,-210)	<0.001	0.578
CT_sd_ value (HU)*	69(46,113)	82(53,124)	98(67,135)	<0.001	76(43,112)	88(51,136)	98(67,138)	0.010	0.829

*Note* BD = bronchial deformation, LD = long diameter, SD = short diameter, CTmax = maximum of CT attenuation value, CTmin = minimum of CT attenuation value, CTmean = mean of CT attenuation value, CTsd = standard deviation of CT attenuation, HU = hounsfield unit.The adoption of statistical values is consistent with Table 1

### Comparison of the efficiency of the four models

**Fig. 4:**
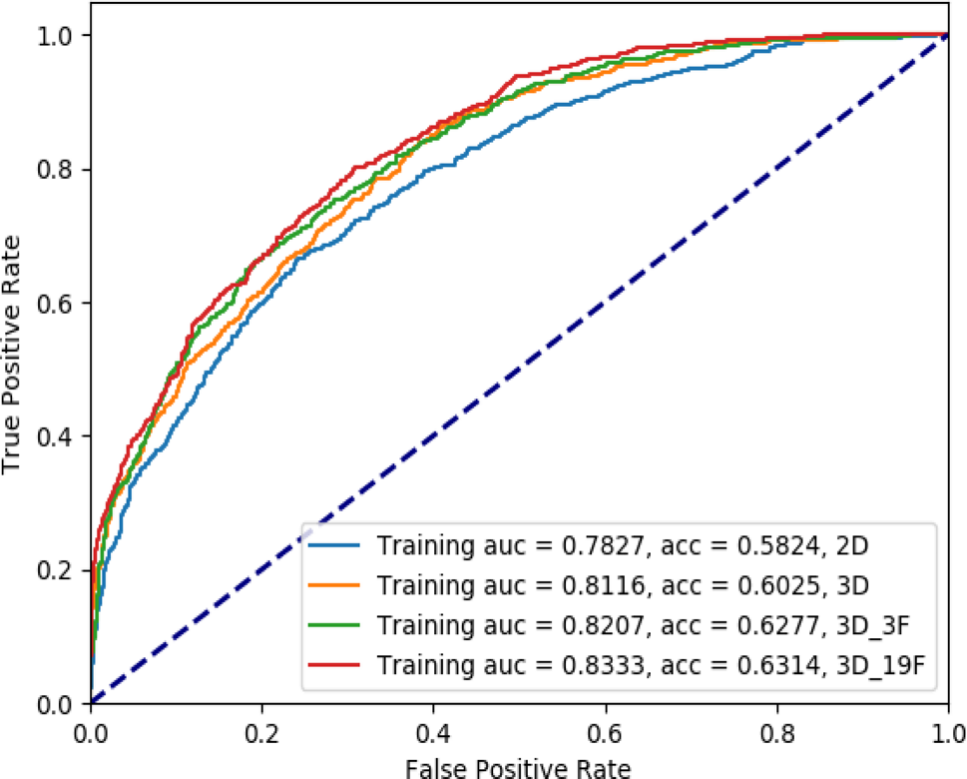
ROC curves for the four models in the training set

Figure 4 presents the ROC curves for the four models in the training set. The AUCs for detecting the invasiveness of pGGNs as a three-category classification were 0.7827, 0.8116, 0.8207, and 0.8333 for the EfficientNet-b0 2D, dual-head ResNet_3D, 3D_3F, and 3D_19F models, respectively. Furthermore, the ResNet_3D_3F model has slightly higher diagnostic efficiency than the ResNet_3D_4F and ResNet_3D_6F models (Supplementary Fig.S7 and S8). As presented in Table 3, statistical differences are detected in diagnostic efficiency between the 2D and 3D models using the Obuchowski index (*P* < 0.05).

**Table 3:** The Obuchowski index results of the four models in training set

Cohort	Model 1	Model 2	Obuchowski index of Model 1	Obuchowski index of Model 2	*P*-value
Training set	EfficientNet-b0 2D	Dual-head ResNet_3D	0.769591	0.813060	0.000045
	EfficientNet-b0 2D	Dual-head ResNet_3D_3F	0.769591	0.821053	0.000001
	EfficientNet-b0 2D	Dual-head ResNet_3D_19F	0.769591	0.846473	<0.000001
	Dual-head ResNet_3D	Dual-head ResNet_3D_3F	0.813060	0.821053	0.159768
	Dual-head ResNet_3D	Dual-head ResNet_3D_19F	0.813060	0.846473	0.000001
	Dual-head ResNet_3D_3F	Dual-head ResNet_3D_19F	0.821053	0.846473	0.001379

**Fig. 5:**
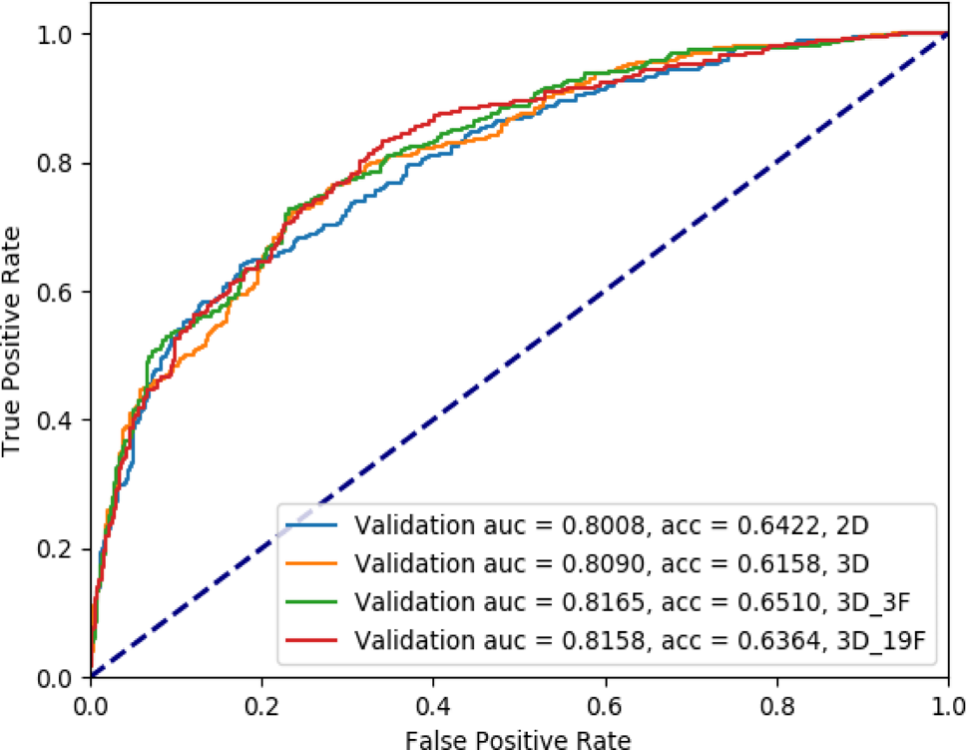
ROC curves of the four models in the validation set

Figure 5 presents the ROC curves of the four models in the validation set. The AUCs for detecting the invasiveness of pGGNs as a three-category classification are 0.80080, 0.80900, 0.81650, and 0.8158 for the EfficientNet-b0 2D, dual-head ResNet_3D, 3D_3F, and 3D_19F models, respectively. Among the four models, the dual-head ResNet_3D_3F model has the highest diagnostic efficiency. Table 4 illustrates that the diagnostic efficiencies of the four models do not differ as tested by the Obuchowski index, but the diagnostic efficiency is higher for the 3D models than for the 2D model.

**Table 4:** The Obuchowski index results of the four models in validation set

Cohort	Model 1	Model 2	Obuchowski index of Model 1	Obuchowski index of Model 2	*P*-value
Validation set	EfficientNet-b0 2D	Dual-head ResNet_3D	0.791149	0.813164	0.104246
	EfficientNet-b0 2D	Dual-head ResNet_3D_3F	0.791149	0.813500	0.111698
	EfficientNet-b0 2D	Dual-head ResNet_3D_19F	0.791149	0.815234	0.143505
	Dual-head ResNet_3D	Dual-head ResNet_3D_3F	0.813164	0.813500	0.970531
	Dual-head ResNet_3D	Dual-head ResNet_3D_19F	0.813164	0.815234	0.860624
	Dual-head ResNet_3D_3F	Dual-head ResNet_3D_19F	0.813500	0.815234	0.881811

**Fig. 6:**
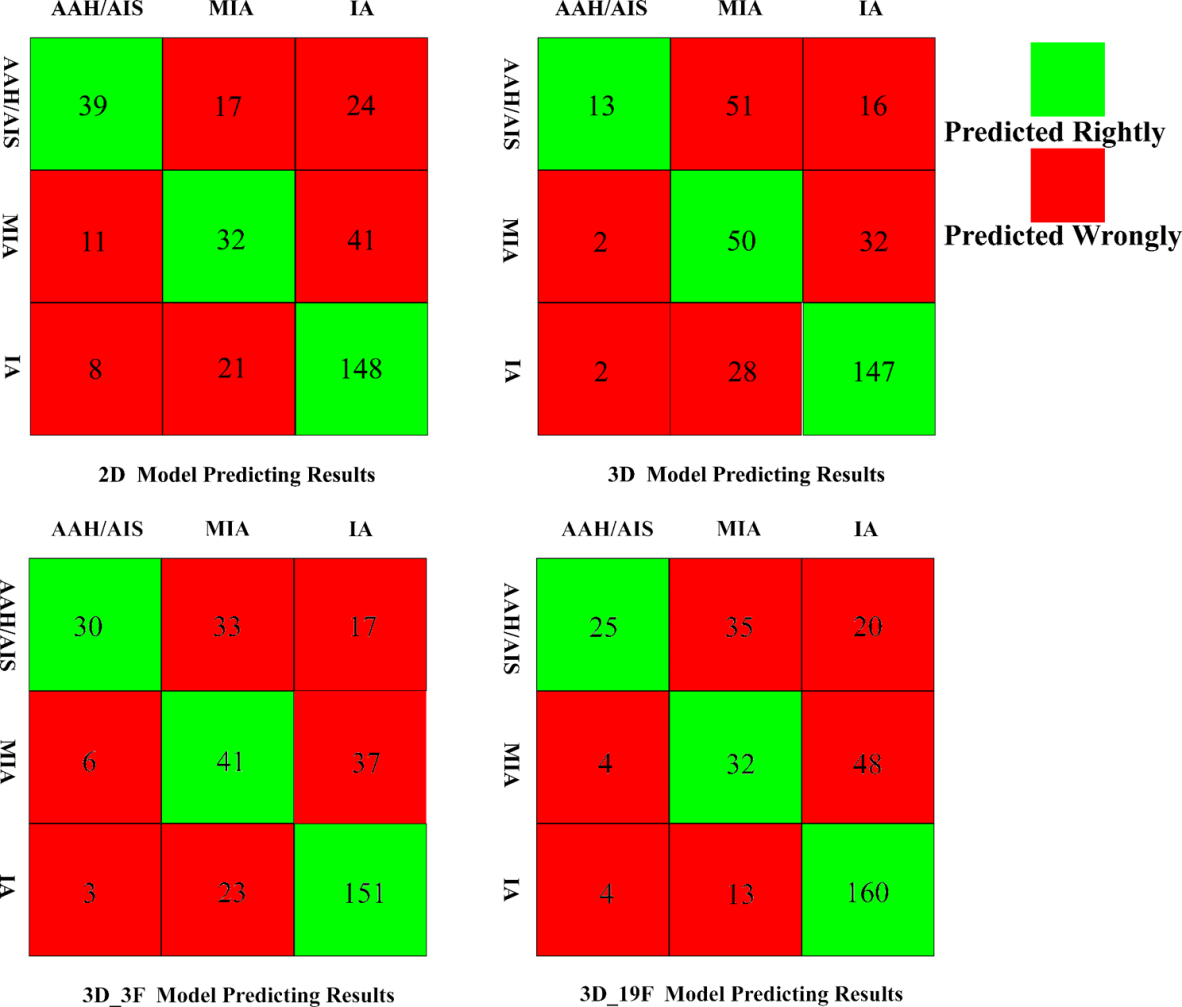
The confusion matrix for the four models

The confusion matrix for each model is presented in Fig. 6. The figure presents the interval diagram of the distribution of the number of three-category classifications predicted by each model. Table 5 shows the diagnostic efficiencies of the four models in the training set and the validation set. Among the four models, the prediction efficiencies in descending order are Dual-head ResNet_3D_3F, Dual-head ResNet _3D _19F, Dual-head ResNet _3D and Dual-head ResNet_2D, respectively.

**Table 5:** Diagnostic efficiencies of four models in training set and validation set

	EfficientNet-b0 2D	Dual-head ResNet_3D	Dual-head ResNet_3D_3F	Dual-head ResNet_3D_19F
Training set				
AUC	0.7827	0.8116	0.8207	0.8333
ACC	0.5824	0.6025	0.6277	0.6314
R	0.5823	0.5989	0.6136	0.6314
P	0.5587	0.6002	0.6034	0.8030
F1	0.5655	0.5639	0.5964	0.6165
Validation set				
AUC	0.8008	0.8090	0.8165	0.8158
ACC	0.6422	0.6158	0.6510	0.6364
R	0.6371	0.6662	0.6511	0.6362
P	0.6263	0.6066	0.6685	0.6405
F1	0.5626	0.5853	0.6442	0.6100

*Note* AUC = Area under the ROC curve, ACC = Accuracy, R = Recall, P = Precision, F1 = F1-Score

## Discussion

To the best of our knowledge, no previous studies used a deep learning technique to specifically evaluate the invasiveness of pGGNs. Therefore, this is the only study to evaluate the invasiveness of pGGNs (three-category classification) using the ResNet network and combining different clinical features. We found that dual-head ResNet_3D_3F model had the highest diagnostic efficiency, whereas there were no significant differences in the diagnostic efficiency compared to other models. In clinical practice, the dual-head ResNet_3D_3F model can be used to evaluate the invasiveness of pGGNs. The results of this study are helpful to intelligently evaluate the invasiveness of pure ground-glass nodules, and provide methodological reference for future related studies.

This study illustrated that the incidence of IAC increases with age, consistent with previous findings [28, 29]. In this group, a mean age exceeding 59 years was useful for categorizing the invasiveness of pGGNs. Some studies revealed that the smoking status is significantly associated with the invasiveness of GGNs [30], contradicting the current findings. It has been reported in the literature that the smoking status is closely related to the growth of lung cancer manifested as GGNs and linked to increased invasiveness [30, 31]. Smokers could be more likely to develop lung adenocarcinoma with subsolid GGNs. In this group, the study population was mainly women, and the overall proportion of smokers was low. These findings could explain the non-significant association of the smoking status with pGGN invasiveness.

Most of the previous literature illustrated that lobulation and spiculation had significance in evaluating the invasiveness of pGGNs [7, 16, 32] and subsolid GGNs [12, 33], in line with the current study findings. However, the utility of the air bronchogram sign in differentiating the invasiveness of GGNs has been inconsistent [29, 33–35]. In this group, all patients with air the bronchogram sign had MIA or IAC, and the air bronchogram sign with deformation was more common in IAC. Therefore, we concluded that the air bronchogram sign with deformation was a strong indicator of IAC. Lesion size and density were closely correlated with the invasiveness of GGNs [15, 16, 34, 36]. Hsu et al. [14] found that LD, SD, and the average diameter had significance in identifying the invasiveness of pGGNs, similar to our findings. CT data are important indicators of the invasiveness of pGGNs [16, 37, 38], in line with the present data. CTsd reflects the difference in tumor internal density, and greater values indicate greater tumor heterogeneity, which has not been reported in other studies.

The detection of microscopic changes and hierarchical features invisible to the human eye is one of the advantages of deep learning, and this helps to better distinguish the subtypes of lung cancer [25, 26, 39, 40]. The present study used 2D and 3D models in addition to models combining different clinical features (3 F, 4 F, 6 F, 19 F), differing from the simple 3D deep learning network models reported in other studies [25–27]. In this cohort, the ACCs of simple 2D and 3D models for predicting the invasiveness of pGGNs (three-category classification) were 0.6422 and 0.6158, respectively, consistent with the results reported by Zhao [25] and Yu [41]. The AUC and ACC for pGGN invasiveness (three-category classification) evaluated by the dual-head ResNet_3D_3F model were 0.8165 and 0.651, respectively, exceeding the values obtained using other models. We found that precision was more important in the evaluation than the number of clinical features combined. In clinical practice, sex, age, and the smoking status are easy to obtain and simple to implement, highlighting their good prospects for use in future clinical practice. In the dual-head ResNet_3D_3F model, the positive prediction efficiency of lung precursor lesions was the highest (76.9%), followed by IAC (73.7%) and MIA (42.2%). Therefore, patients with lung precursor lesions benefited the most from this model, which can effectively alleviate the anxiety of these patients; Secondly, the benefit was IAC, which required shorter follow-up time or elective surgery.

The ResNet network was used in this study, and it adopted a multi-channel residual connection in the residual block in place of a single-channel residual connection [42]. In this manner, the ResNet network can obtain fine-grained multi-scale features and increase the receptive field of the network. This study differed from prior research by improving multi-scale capability through the use of different resolutions [43, 44]. At the same time, the ResNet module can be inserted into other classic backbone networks such as ResNet [45], ResNeXt [46], and DLA [47] to achieve better results. The dual-head ResNet model adopted in this study first extracts the feature vectors of medical images using the ResNet network and uses the fully connected network to classify the feature vectors, and finally, the classification results obtained by the dual-head model are weighted and combined to obtain the final classification results to obtain better results. Meanwhile, the simple 2D model uses the EfficientNet-B0 network model, the core structure of which is the mobile inverted bottleneck convolution module [48].

Our study had multiple limitations. First, the ResNet network used in this study is relatively simple. It was not compared with other networks, such as ResNet, UNet, and DenseNet, nor was it integrated with other networks. Further research is necessary. Second, external validation was not introduced, which may affect model generalization. Next, we will actively collaborate with more hospitals to conduct external validation to determine the generalization of the model. Finally, the weak explanation of deep learning techniques is also a limitation of this study. The internal mechanisms of deep learning systems should be explored in future studies.

## Conclusion

Four different models evaluating pGGNs invasiveness (three-category classification) have good diagnostic efficacy, in which dual-head ResNet_3D_3F model had the highest diagnostic efficiency. In clinical practice, the dual-head ResNet_3D_3F model can be applied to evaluate pGGN invasiveness, thereby providing a valuable tool for accurate diagnosis in patients.

### Electronic supplementary material

Below is the link to the electronic supplementary material.

Supplementary Material 1

Supplementary Material 2

Supplementary Material 3

## Data Availability

The datasets used and/or analysed during the current study are available from the corresponding author on reasonable request.

## Abbreviations

AAH: Atypical adenomatous hyperplasia
AIS: Adenocarcinoma in situ
MIA: Minimally invasive adenocarcinoma
IAC: Invasive adenocarcinoma
CT: Computed tomography
pGGN: Pure ground glass nodule
ACC: Accuracy
AUC: Area under curve
ResNet: Residual Network
