# Supplementary figures and images for: Evaluation of the invasiveness of pure ground-glass nodules based on dual-head ResNet technique
Source: BMC Cancer. 2024 Sep 2;24:1080. doi: 10.1186/s12885-024-12823-4 (PMC11367849; doi:10.1186/s12885-024-12823-4)

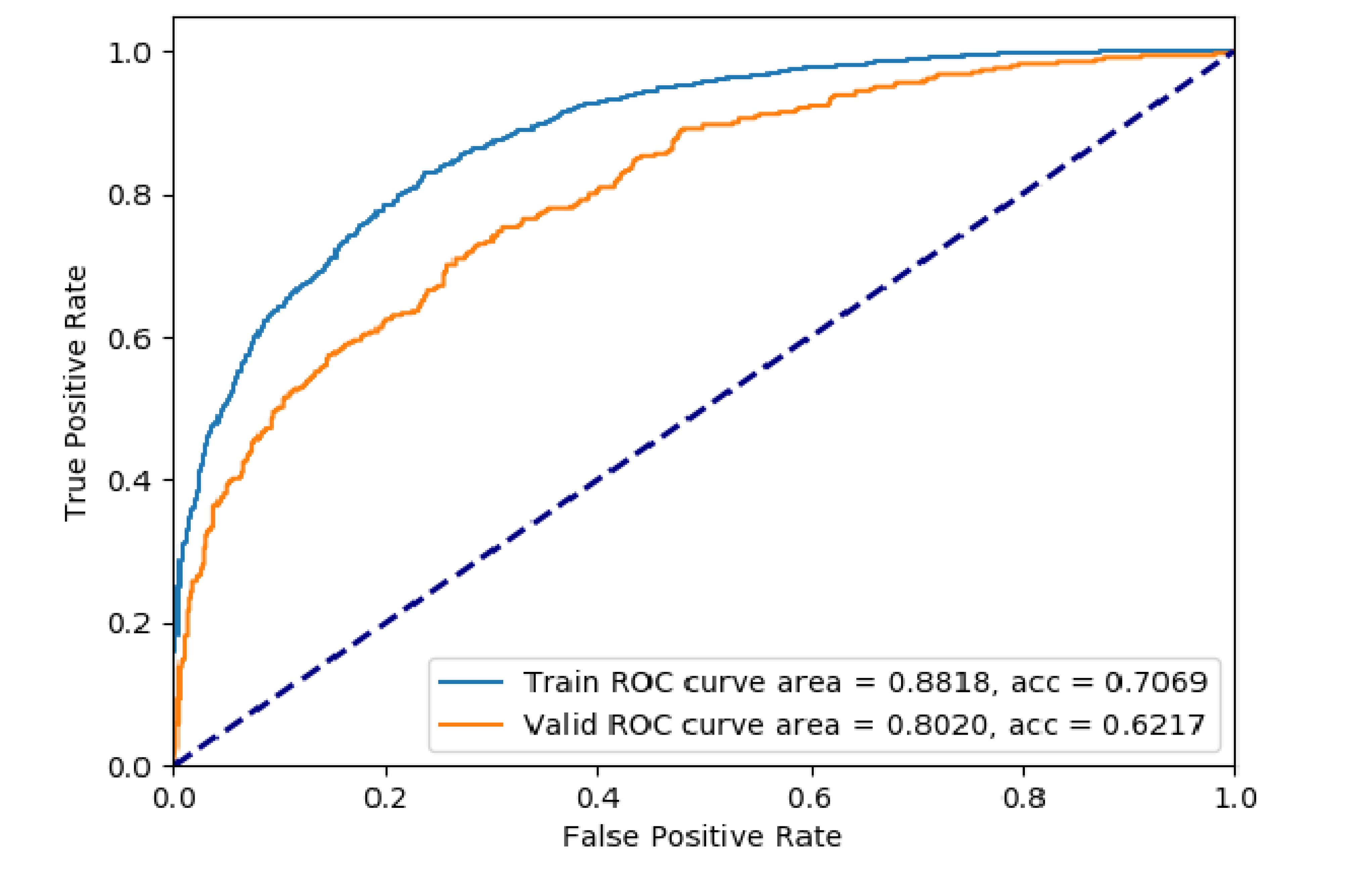

Supplement: Supplementary file 1 — Supplementary Material 1 [file 12885_2024_12823_MOESM1_ESM.tif]
