# Supplementary material for: Evaluation of the invasiveness of pure ground-glass nodules based on dual-head ResNet technique
Source: BMC Cancer. 2024 Sep 2;24:1080. doi: 10.1186/s12885-024-12823-4 (PMC11367849; doi:10.1186/s12885-024-12823-4)
Supplement: Supplementary file 3 — Supplementary Material 3 [file 12885_2024_12823_MOESM3_ESM.docx]

**The specific image processing and model construction is as follow：**

Training was performed by running on the Ubuntu18.04 with Intel(R) Xeon(R) Gold 6226R CPU (2.90 GHz), 256 GB RAM, and NVIDIA Tesla V100 GPU with 16 GB of memory.

In this study, the EfficientNet-b0 and dual-head ResNet networks were used. First, all CT images were homogenized. The 2D model divides the mask according to the 3D coordinates of the focal point, selects the maximum mask layer, uses an intensity window range of [−600, 1500], maps it to an intensity range of 0–255, interpolates it to [32 × 32 × 32], and maps it to the range of 0–1 after multiple rounds of data enhancement. The Efficient Net-b0 model was fed for training.

The entire structure of the 3D model dual-head Res2Net network is presented in **Fig.2**. The first part of the 3D model processing step is the same as the 2D model, until the data is mapped to the range of 0–1, and then blocks of [32 × 32 × 16] and [32 × 32 × 8] were cut from the [32 × 32 × 32] block and fed to the dual-head ResNet model for training.

The [32 × 32 × 16] and [32 × 32 × 8] blocks were entered into the dual-head ResNet model, which was passed through eight consecutive ResNet bottleneck modules and n full connection layers. Four types of results, namely out1_1, out1_2, out2_1, and out2_2, were obtained, two of which were outputted directly for concatenation and the remaining two were combined with clinical features for concatenation. After the full connection layer was obtained, the predicted scores of out1 and out2 in each category were outputted. The softmax operation was performed on out1 and out2 to obtain the probability of each category, and the probability was weighted at a 2:1 ratio (namely 0.667 × out1 + 0.333 × out2). The final prediction probability was obtained via weighting. The model uses the cross-entropy loss function.
